# Almost Everyone Loses Meaning in Life From Social Exclusion, but Some More Than the Others: A Comparison Among Victims, Voluntary, and Forced Rejecters

**DOI:** 10.3389/fpsyg.2021.658648

**Published:** 2021-07-07

**Authors:** Shuyue Zhang, Junqing Huang, Hedan Duan, Ofir Turel, Qinghua He

**Affiliations:** ^1^Department of Psychology, Faculty of Education, Guangxi Normal University, Guilin, China; ^2^Guangxi University and College Key Laboratory of Cognitive Neuroscience and Applied Psychology, Guangxi Normal University, Guilin, China; ^3^Ethnic Education Development Research Center of Guangxi Zhuang Autonomous Region, Guilin, China; ^4^Information Systems and Decision Sciences, California State University, Fullerton, CA, United States; ^5^Ministry of Education Key Laboratory of Cognition and Personality, Faculty of Psychology, Southwest University, Chongqing, China

**Keywords:** social exclusion, ostracism, meaning in life, rejecter, motivation

## Abstract

Social exclusion has been a major societal concern because it hinders the attainment of needs for belonging and relationship. While we know much about the effects of social exclusion on victims and perpetrators, there is limited insight regarding how different types of rejecters (voluntary vs. forced) might affect important outcomes. The purpose of this study is to bridge this gap and to examine how different types of social exclusion (forced and voluntary) influence meaning in the life of participants. To this end, we conducted two experiments using two social exclusion paradigms: the recall paradigm and the Cyberball game. The results of the two experiments were consistent. Both experiments revealed that (1) the meaning in the life of the victim group and the forced rejecter group (i.e., those who were forced to exclude others) was significantly lower than this of the control group and the voluntary rejecter group (i.e., those choosing to exclude others). There were no significant differences between the victim group and the forced rejecter group, and there were no significant differences between the voluntary rejecter group and the control group. These results reveal that social exclusion not only negatively affects the victims of exclusion but also reduces the meaning in the life of forced rejecters. These findings are specific, and they show that the types of will in exclusion can create differences in the effects of social exclusion on the rejecters.

## Introduction

Social exclusion is the phenomenon and process that the needs of a person for belonging and relationship are hindered due to being rejected or excluded by someone or a social group (MacDonald and Leary, 2005; Williams, 2007). It has been prevalent throughout history and existed in various social status groups and cultures (Byrne, 2005). Its importance and prevalence stem from the idea that humans are social animals (Frith and Frith, 2012). They depend upon social relationships to fortify their physical and psychological well-being (Wesselmann and Williams, 2017). This means that establishing and maintaining social connections with others is one of the strongest human needs (Abraham, 1943). Social exclusion is a threat to this demand and prevents the attainment of human social needs. According to Williams' ostracism model, being ostracized impairs multiple psychological needs, including the need for a meaningful existence (e.g., belongingness, self-esteem, and control) (Williams, 2009; Lansu et al., 2017; Sandstrom et al., 2017). Because the social connection is a biological strategy ingrained in human, being cut off from others raises the potential threat of losing access to all socially mediated meanings, purposes, and values (Stillman et al., 2009). Consequently, social exclusion increases the sense of helplessness and worthlessness, which in turn, threatens the meaning in the life of people.

Meaning in life encapsulates the beliefs of people about their life purposes, goals, and missions as derived from reflecting on themselves, the world around them, and their position in the world (Steger et al., 2009, 2015; Steger, 2017). People with higher meaning in life have lower stress (Mascaro and Rosen, 2006), higher levels of psychological well-being and health (Zika and Chamberlain, 1992; Hooker et al., 2018); they are also focused on contributing more to society (Bonebright et al., 2000; Klein, 2017). In contrast, people with low meaning in life were more likely to exhibit problematic behaviors, such as suicide (Kleiman and Beaver, 2013; Heisel et al., 2016), overeating (García-Alandete et al., 2018), illegal drug abuse (Steger et al., 2015), and alcoholism (Ostafin and Feyel, 2019). Therefore, meaning in life is important for individuals and society. Previous studies have found that both temporary exclusion in an experiment (Stillman et al., 2009) and real-life exclusion (Jiang and Chen, 2020) can cause a decline in meaning in the life of an individual.

The previous research on social exclusion has been mostly focusing on victims of social exclusion and investigated the outcomes of such exclusions (MacDonald and Leary, 2005; Zadro and Gonsalkorale, 2014; Grahe, 2015). However, social exclusion involves two types of people with different roles: the perpetrator of the exclusion (i.e., the rejecter, sources of exclusion), and the victim of exclusion (i.e., the person being excluded by others). Therefore, a gap exists in the understanding of the impact of social exclusion on both parties. How the effect on the rejecter might differ from the victims? This study aims at making strides toward addressing this gap. It specifically compared the levels of meaning in life between the two parties involved in social exclusion.

We note that previous studies have produced inconsistent results about the effects of social exclusion on the rejecter (perpetrators of exclusion). Some studies suggest that social exclusion has a positive effect on the rejecter, such as making the relationship among rejecters closer and stronger (Wyer and Schenke, 2016). Other studies suggested that social exclusion not only hurts victims but also drives painful feelings in rejecters (Chen et al., 2014). For instance, exclusion behaviors increase rejecters' sense of guilt and embarrassment (Poulsen and Kashy, 2012). Rejecters have the most negative emotions (mainly guilt and shame) and the lowest autonomy and relatedness (Legate et al., 2013). Their sense of belonging was decreased after excluding others (Gooley et al., 2015; Nezlek et al., 2015).

These studies have one important limitation: They have lumped together different motivations for social exclusion. In some studies (mostly experimental), the exclusion behavior of the rejector was involuntary. Instead, it was requested by the experimenter (Poulsen and Kashy, 2012; Legate et al., 2013). In other studies, researchers just asked participants to report their experience of excluding others but did not distinguish the motivations of actions of participants (Nezlek et al., 2015). It means that the action of exclusion may not be done by rejecters willingly. According to the self-determination theory (SDT) (Ryan and Deci, 2000), when basic psychological needs for autonomy, capability, and relatedness are met, people flourish; when these needs are not met, people may suffer and respond defensively. Different motivations for social exclusion (voluntary vs. forced) may, therefor, lead to different or contradictory effects on the perpetrator.

To examine this possibility, we defined social exclusions made at the request of others (involuntary) as forced exclusion and behaviors of exclusion that were made under one's own will (self-determined) as voluntary exclusion. Given that the adverse impacts of social exclusion tend to be larger in victims than in perpetrators (Zadro et al., 2004), study assumed that meaning in life would be different due to the different roles in the social exclusion process. We assume the following: (a) the voluntary rejecter would have higher scores of meaning in life than the forced rejecters and the victims of exclusion; and (b) the victims of exclusion would have lower levels of meaning in life as compared with the rejecters group and the control group.

We test these assertions with two experiments, using different paradigms: the recall paradigm and the Cyberball game. In both experiments, we use between-subjects designs. Participants were divided into four groups: (1) victim group, (2) voluntary rejecter group, (3) forced rejecter group, and (4) control group. In conclusion, this article clarifies whether different motivations behind social exclusion bring about different effects of social exclusion on meaning in life.

## Materials and Methods

### Experiment 1

*Participants:* Following Jiang's (2020) experiment, we recruited 192 undergraduate students by using a class announcement in a psychology course. Twelve participants who did not finish the whole experiment were dropped. We consequently retained *n* = 180 (131 females, *M*_*age*_ = 18.35, *SD*_*age*_ = 1.77). All procedures were reviewed and approved by the local Institutional Review Board.

*Classification into social exclusion role category:* Division of participants into the four social exclusion role categories (a. victims, b. forced rejecters, c. voluntary rejecters, and d. control group) was based on a sequence of screening questions. It resulted in four equal groups, each with n = 45. Participants entered the laboratory one by one and were requested to answer one of three questions. The first set of the participants were asked “Do you have any experience of being excluded?” Those who answered “Yes” entered the victim group, and those who answered “No” entered the control group. When the number of valid participants in the victim group reached 45, the next participant who entered the laboratory would be asked another question: “Do you have any experience of being compelled to exclude others?” Those who answered “Yes” entered the forced rejecter group and those who answered “No” entered the control group. The same as the victim group, when the forced rejecter group reached 45 participants, the next participant would be asked another question: “Do you have any experience of excluding others based on your own free will?” Those who answered “Yes” entered the voluntary rejecter group. Those participants who answered “no” entered the control group. But when the control group reached 45 participants, the participants who answered “No” were told that their experiment is terminated.

*Meaning in life:* We used the Meaning in Life Questionnaire-presence [MLQ-P (Steger et al., 2006), the five-item scale, translated into Chinese] to measure the meaning in life of participants. Sample items for the present-meaning subscale include the following: “I understand my life's meaning,” “My life has a clear sense of purpose,” “I have a good sense of what makes my life meaningful,” “I have discovered a satisfying life purpose,” and “My life has no clear purpose (revered).” The participants used a seven-point scale (1 = absolutely untrue; 7 = absolutely true) to indicate how typical is each of the 5 items of them. The scale was reliable (α = 0.81). Hence, the sum of item scores was used as a meaning in life index in further analyses. Higher scores indicated higher meaning in life.

*Memories of social exclusion:* We distributed a blank paper to every participant and asked them to write down their memories of the social exclusion which they mentioned in the answer.

#### Procedure

This experiment followed a single factor between-subjects design. The factor (social exclusion role) had four categories: (1) victims, (2) forced rejecters, (3) voluntary rejecters, and (4) control group. The dependent variable was the meaning in life score. The experimenter required participants to come to a quiet laboratory one by one. First, participants were asked to complete the screening question. Then, participants who entered the victim group, the forced rejecter group, and the voluntary rejecter group were asked to recall and write down relevant exclusion memories in as much detail as possible. In the control group, participants wrote what they did the previous day (Park and Baumeister, 2015). When participants finished writing, they completed the meaning in life scale.

*Manipulate check*. After participants finished the writing, the experimenter inspected the text, which was written by participants. Participants who did not write complete and relevant memories, were excluded. For example, participants in the victim group were expected to report a personal incident in which they had felt rejected or left out by others.

#### Results

**Figure 1:**
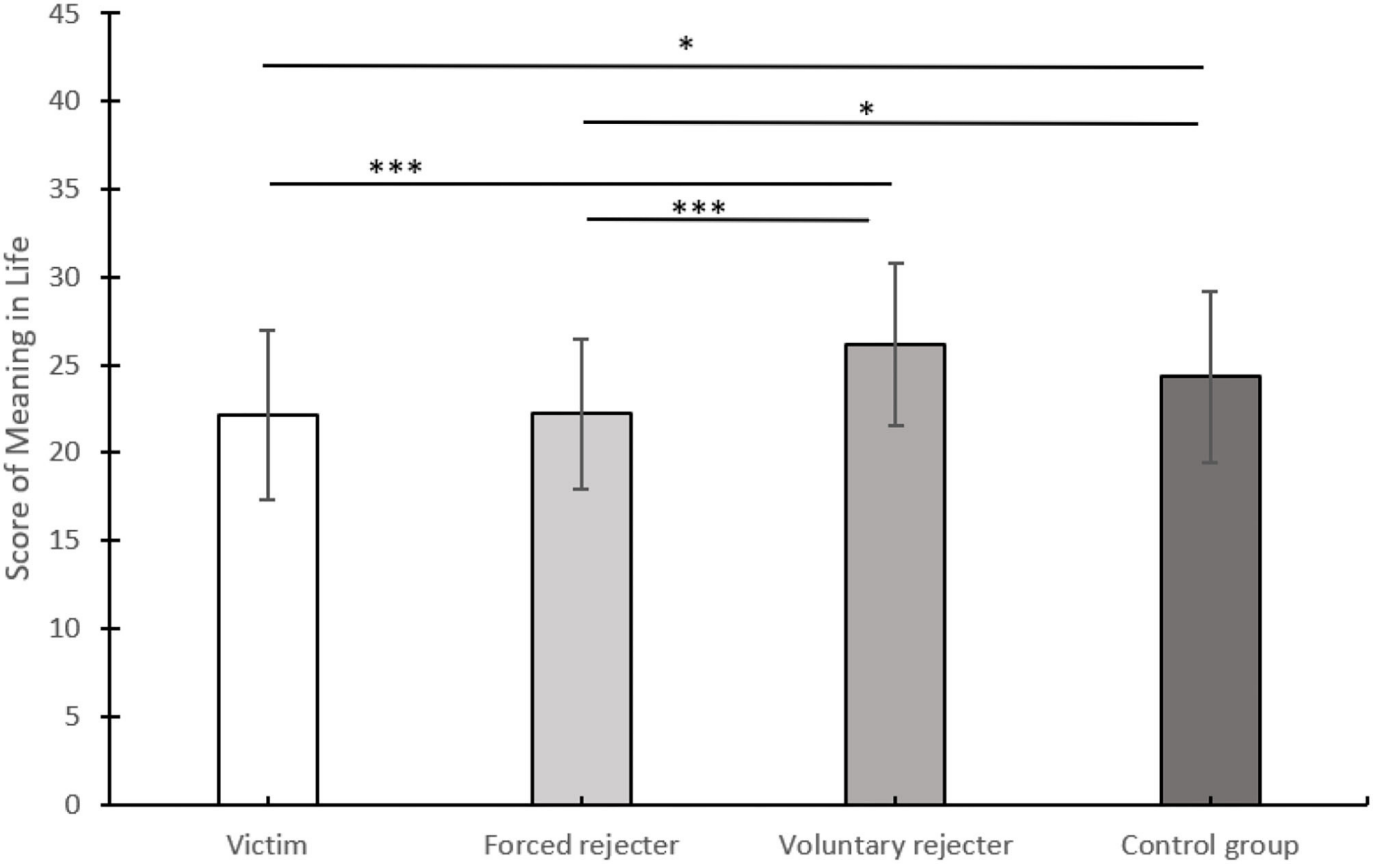
Differences in meaning in life scores among the four groups, experiment 1†. †*p < 0.05, ***p < 0.001.

One-way ANOVA results showed that the four groups of participants had significant differences in meaning in life scores, *F*_(3, 176)_ = 6.82, *p* < 0.001, η*2* = 0.10. *Post-hoc* tests (LSD) showed that the meaning in life of the victim group (*M* = 22.16 and *SD* = 4.84) was significantly lower than this of the control group (*M* = 24.33 and *SD* = 5.90), *p* = 0.038 and the voluntary rejecter group (*M* = 26.20 and *SD* = 4.64), *p* < 0.001. The forced rejecter group score (*M* = 22.24 and *SD* = 4.26) was significantly lower than this of the control group, *p* = 0.047 and the voluntary rejecter group, *p* < 0.001. There was no significant difference between the voluntary rejecter group and the control group, *p* = 0.075. There was no significant difference between the victim group and the forced rejecter group, *p* = 0.932. The results are depicted in Figure 1.

### Experiment 2

Experiment 1 demonstrated that social exclusion not only affects the victims negatively but also affects rejecters. The forced rejecters have been negatively affected by social exclusion similar to the victims. To extend these findings and distinguish the different roles of social exclusion, we use the Cyberball paradigm in experiment 2.

*Participants*. We recruited 241 undergraduate students with an online announcement. 41 problematic records were dropped, which left us with an operational sample of n = 200 (175 females, *M*_*age*_ = 18.64, and *SD*_*age*_ = 0.99). All participants were right-handed, had no recent medical issues, and had normal vision. All procedures were reviewed and approved by the local Institutional Review Board. Participants were randomly divided into four experimental groups, with 50 people in each group. Each group was *a-priori* assigned a different social exclusion role.

*Meaning in life*. Meaning in life scale was identical with experiment 1. In experiment 2, α = 0.87.

*Social exclusion*. In this experiment, the Cyberball paradigm (using the desktop version) was used to distinguish different roles in social exclusion, including the victim group, the forced rejecter group, the voluntary rejecter group, and the control group. Cyberball is a computerized ball-tossing game (Williams et al., 2000). All participants were told that the experiment was about mental visualization during a computer ball-tossing game played with other real players located in another laboratory room. But in fact, the “other players” were manipulated by a computer program (Legate et al., 2013). The specific operation of Cyberball was different in each group. Explanations for how roles were operationalized are given in the “Procedure” section.

*Manipulation check questions for Cyberball*. In the victim group, we asked participants to respond to (yes/no) the question: “I felt I was being rejected in the game.” In the forced rejecter group and the voluntary rejecter group, the question was: “In the earlier game, I rejected other players.” Participants should respond either yes or no. Responses were considered valid only if they answered “yes.”

#### Procedure

Experiment 2 followed a single factor between-subjects design. The factor was the social exclusion role. Similar to experiment 1, this study used the four categories [(1) victim group, (2) forced rejecter group, (3) voluntary rejecter group, and (4) a control group]. In this experiment, the plan was to recruit for each group 45 participants, but this number increased to 50 in the formal experiment. Participants were required to come to a quiet laboratory one by one, just like experiment 1 and, then, they entered one of the four groups randomly. The experiment started with the desktop version of the Cyberball game. Participants were told that it was an online ball-tossing game. They will play with two other real players who were placed in other laboratories (actually, the other players were not real people, it was just a computer program). Then, a “loading…” message appeared for 5 s on the computer after participants opened the program. It mimicked the experience of waiting for others to join the game. Next, the participants took part in the “online” ball-tossing game. The four groups of social exclusion roles operated differently as described below.

In the victim group, the operationalization was the same as Williams' study 2000. The total number of passes was 30. Participants in the victim group only received two passes at the beginning of the game and never received a pass again.

In the forced rejecter group, the operation was the same as Legate et al. (2013) and Bastian et al. (2013). The total number of passes was 30. Before the start of the game, participants were informed that they should not pass the ball to a specific player. For example, when the participant was player A, he or she may receive instructions to exclude player B, they have just been allowed throwing the first two balls to player B, and then, they should only throw to player C. Meanwhile, player C (manipulated by a computer program set by the experimenter) also just throws two balls to player B at the beginning. The experimenters declare that the participants have the right to refuse to do so and quit the experiment.

In the voluntary rejecter group, the operation was the same as the procedure in Wesselmann's et al. (2013) experiment. The total number of passes was 30. Participants in this group were told that they need to finish the game with the other two players as soon as possible. The experimenter would set up a computer-controlled player who waited for 16 s before each pitch (slow speed), while another player passed the ball immediately after receiving the ball (normal speed). The slow-speed player increased the waiting time of passing. This was expected to increase impatience of the participant, which induces voluntary social exclusion: reduces the number of passing to the slow player. In addition, the normal-speed player did not throw the ball to the slow-speed computer player and only passed the ball to the participant.

In the control group, participants randomly received the ball during the game. They had the same chance of receiving from each other players (the number of balls that participants received was one-third of the total pass (*n* = 10 = 33.33% ^*^ 30). Participants could pass the ball to player A or player B freely.

After the Cyberball game, all participants completed the manipulation check questions and the Meaning in Life Questionnaire-presence.

#### Results

**Figure 2:**
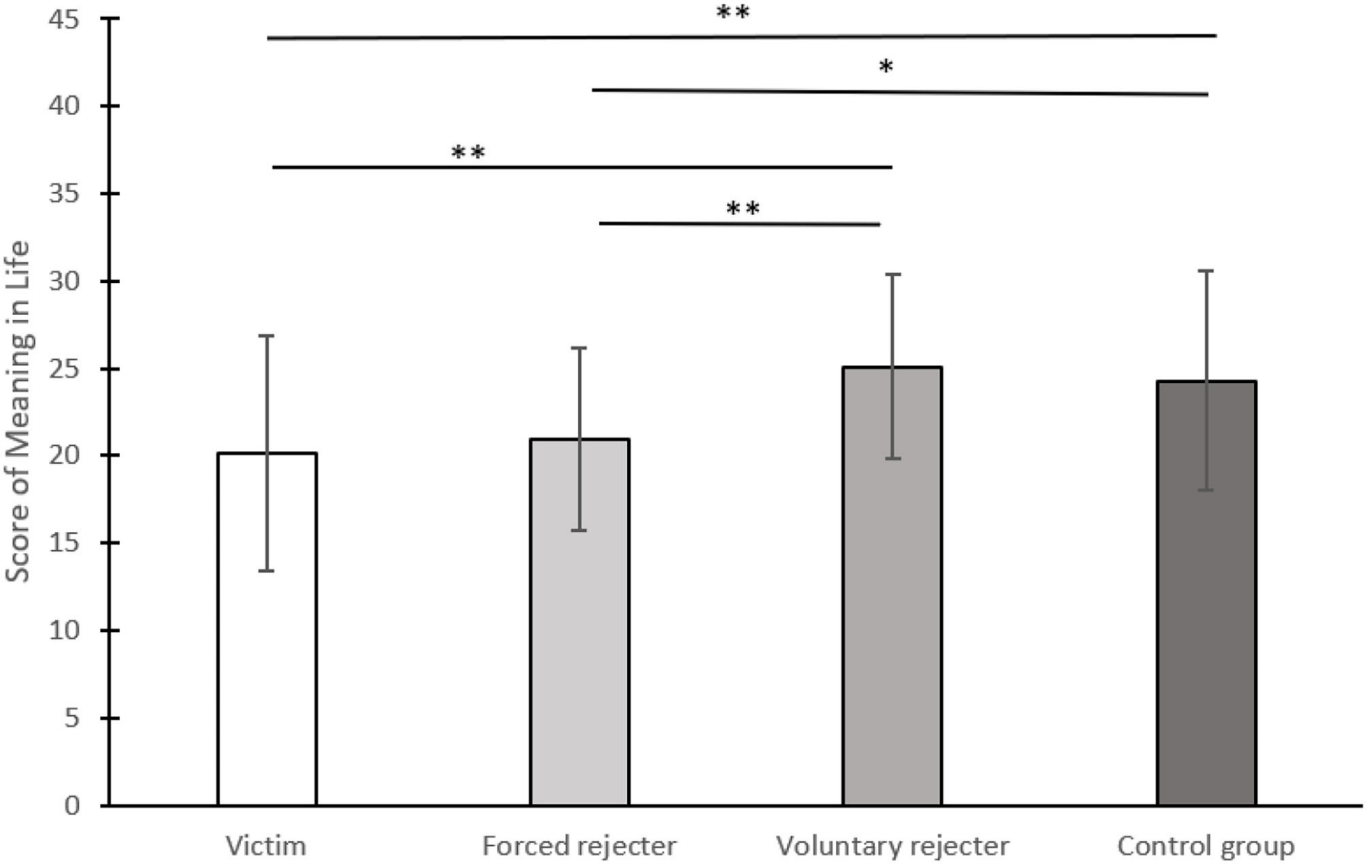
Differences in meaning in life scores among the four groups, experiment 2†. †*p < 0.05, **p < 0.01.

The meaning in life score was considered as the dependent variable. One-way ANOVA showed that the four groups had significant differences in meaning in life scores, *F*_(3, 196)_ = 8.43, *p* < 0.001, η^2^ = 0.11. In this analysis, we used more conservative methods, Tukey-HSD for *post-hoc* tests. The results showed that the meaning in life score of the victim group (*M* = 20.18, *SD* = 6.73) was significantly lower than this of the control group (*M* = 24.28, *SD* = 6.26), *p* = 0.004 and the voluntary rejecter group (*M* = 25.10, *SD* = 5.25), *p* < 0.001. The forced rejecter group (*M* = 20.96, *SD* = 5.23) had significantly lower meaning in life scores compared with the control group, *p* = 0.028, and the voluntary rejecter group, *p* = 0.003. The meaning in the life of the voluntary rejecter group has no significant difference from the control group, *p* = 0.89. The victim group and the forced rejecter group were also not significantly different, *p* = 0.91. These results are depicted in Figure 2.

## Discussion

This study sought to extend prior research. We focus on the source and the victim of social exclusion simultaneously while distinguishing two types of social exclusion by the motivation of the rejecter: voluntary or forced. To address these objectives, this study reports two experiments that used different paradigms. This approach was taken to increase the generalizability of findings across paradigms because the use of different paradigms can affect results in social exclusion research (Bernstein and Claypool, 2012). In this article, in both studies, we compared the meaning in life scores among four groups reflecting different social exclusion roles: (1) victims, (2) forced rejecters, (3) voluntary rejecters, and (4) a control group (neither experience as a victim nor as a rejecter). In experiment 1, we used the recall paradigm to assign people into four social exclusion role groups. In experiment 2, we randomly assigned people to these four groups and, then, used the Cyberball game paradigm to induce the sense of exclusion. The results of both studies were consistent and supported the assertions: the impact of social exclusion on meaning in life can vary according to the different roles in the process of social exclusion. It shows that almost every person lost meaning in life from social exclusion. This negative relationship involved both victims and rejecters. But the extent of this effect was different between the victims and rejecters.

In experiment 1, the recall paradigm was used to distinguish different roles in social exclusion. For the rejecters, the results showed that the meaning in the life of the forced rejecter group was significantly lower than this of the voluntary rejecter group and the control group. In experiment 2, we changed the recall paradigm to the Cyberball game paradigm. The result was consistent with experiment 1. Together, the results of experiments 1 and 2 reveal that social exclusion not only has a negative effect on the victims but also has an effect on the forced rejecters. These findings are consistent with some previous research (Legate et al., 2013; Chen et al., 2014; Wyer and Schenke, 2016). In the voluntary rejecter group, the score of meaning in life has no significant difference from the control group; however, they were significantly higher than this of the forced rejecter group. This result supports the self-determination theory (Ryan and Deci, 2000). Although forced rejecters were not victims of social exclusion, their act of exclusion does not stem from their own willingness. It damaged their control of the personal relationship, which means the needs for autonomy and relatedness were impaired, considering that people have an inherent tendency to determine their behavior (Legate et al., 2015). When this principle was violated, they developed resistance in form of reactance (Brehm, 1966), which can significantly reduce their meaning in life scores.

This study findings show that volition in exerting exclusion can create differences in the effects of the exclusion on the rejecters. Based on social identity theory, such volition numbs the pain which rejecter may feel under other circumstances and make them having meaning in life scores equivalent to those observed in a control group. In other words, it is possible that having self-determined decisions to exclude others provides the needed mental justification to not feel remorse about such actions, or self-affirmation, and as such, voids the negative effects on the self of perpetrating social exclusions on others. This may explain why the effects of social exclusion on the rejecters were inconsistent in prior studies. This is due to lumping together different motivations for exclusion.

For the victim of social exclusion, suffering from social exclusion has led to a lower meaning in life. The results were consistent in both experiments and it is similar to prior findings (Stillman et al., 2009). Previous studies have found that the experience of stressful life events was associated with meaning in life (Debats et al., 1995; Machell et al., 2015). It may cause people to feel frustrated, and in turn, affect the positive experience of an individual and reduce the meaning in the life of an individual. Regardless of whether the person is the victim of exclusion or a forced rejecter, social exclusion threatened their ability to meet needs for socialization and autonomy.

In conclusion, prior studies mostly focused on the victim of exclusion. But it is also important to study the psychological effects on rejecters in social exclusion. It can help researchers understand how exclusion behavior happened and its effects. This research reveals that the rejecter (source of exclusion) may also be negatively affected by exclusion: the meaning in the life of forced rejecters has a similar reduction as the victims, which means, the bond between perpetrator and victim may not be very clear. The rejecter can also become the victim of social exclusion when their exclusion behavior is involuntary. So the different motivations and autonomy to decide of rejecters may explain inconsistent results in past research. Meaning in life plays an important role in personal growth and daily life. Low meaning in life leads to psychological distress, manifested in emotional problems and suicidal thoughts (Li et al., 2019). Therefore, these findings suggest that we should pay attention to the negative effects which both victims and forced rejecters suffer in social exclusion.

Several limitations of this study should be acknowledged. First, meaning in life is the only outcome of exclusion we observed. Subsequent research can extend this work by focusing on many other potential outcomes of exclusion. Second, while we observed the effects of exclusion on meaning in life, we did not measure the mechanisms that lead to such effects (e.g., reactance, perceived volition, etc.). Future research can directly examine the internal mechanisms that underlie the observed effects. Finally, most of the participants in this study were female. It may affect the generalizability of our results.

## Conclusion

Overall, social exclusion has a negative effect on meaning in life, and this effect varies between different roles in the social exclusion process. For the forced rejecters and the victims, psychological needs for autonomy and relatedness were not met, so that, meaning in the life of these two groups were impaired by social exclusion. In addition, we did not find this negative effect in the voluntary rejecter group. It showed that autonomy is an important factor in social exclusion.

## Data Availability Statement

The raw data supporting the conclusions of this article will be made available by email the corresponding author with reasonable request.

## Ethics Statement

The studies involving human participants were reviewed and approved by IRB of Guangxi Normal University. The patients/participants provided their written informed consent to participate in this study.

## Author Contributions

HD performed the material preparation and data collection. SZ performed the data analysis. JH wrote the first draft of the manuscript. QH and OT revised and improved the quality of the analyses and critically revised the draft. All authors contributed to the conception, design of the study, and read and agreed to the published version of the manuscript.

## Conflict of Interest

The authors declare that the research was conducted in the absence of any commercial or financial relationships that could be construed as a potential conflict of interest.
